# Development and Validation of a Simple UV–HPLC Method to Quantify the Memantine Drug Used in Alzheimer’s Treatment

**DOI:** 10.3390/ph17091162

**Published:** 2024-09-02

**Authors:** Débora Nunes, Tânia G. Tavares, Frenacisco Xavier Malcata, Joana A. Loureiro, Maria Carmo Pereira

**Affiliations:** 1 LEPABE—Laboratory for Process Engineering, Environment, Biotechnology and Energy, Faculty of Engineering, University of Porto, Rua Dr. Roberto Frias, 4200-465 Porto, Portugal; 2 ALiCE—Associate Laboratory in Chemical Engineering, Faculty of Engineering, University of Porto, Rua Dr. Roberto Frias, 4200-465 Porto, Portugal

**Keywords:** HPLC method development, HPLC method validation, high-performance liquid chromatography, Alzheimer’s drug, 9-fluorenylmethyl chloroformate, lipid nanoparticles

## Abstract

Memantine, a non-competitive NMDA receptor antagonist, is used to treat Alzheimer’s disease. Therefore, loading memantine in nanoparticles (NPs) could be an essential tool to improve the treatment effectiveness while reducing drug toxicity. Even though some approaches have been described to quantify memantine, none reported optimized methods using high-performance liquid chromatography resorting to ultraviolet detection (UV–HPLC) to determine encapsulation in NPs. The present research developed a HPLC method using pre-column derivatization for quantitatively analyzing memantine hydrochloride in NPs. Memantine was derivatized using 9-fluorenylmethyl chloroformate (FMOC). The developed method was fully validated regarding suitability, specificity, linearity, sensitivity, precision, accuracy, and robustness according to the International Conference on Harmonisation of Technical Requirements for Registration of Pharmaceuticals for Human Use guidelines. The retention time of memantine was 11.393 ± 0.003 min, with a mean recovery of 92.9 ± 3.7%. The new chromatographic method was validated and found to respond linearly over 5–140 μg/mL, with a high coefficient of determination. Intraday precision lay between 3.6% and 4.6%, and interday precision between 4.2% and 9.3%. The stability of memantine was also tested at 4 °C and −20 °C, and no signs of decay were found for up to 6 months. The new method was properly validated and proved simple, sensitive, specific, accurate, and precise for determining memantine encapsulation efficiency in lipid NPs. Greenness was evaluated, presenting a final score of 0.45. In the future, this methodology could also be applied to quantify memantine in different nanoformulations.

## 1. Introduction

**Figure 1:**
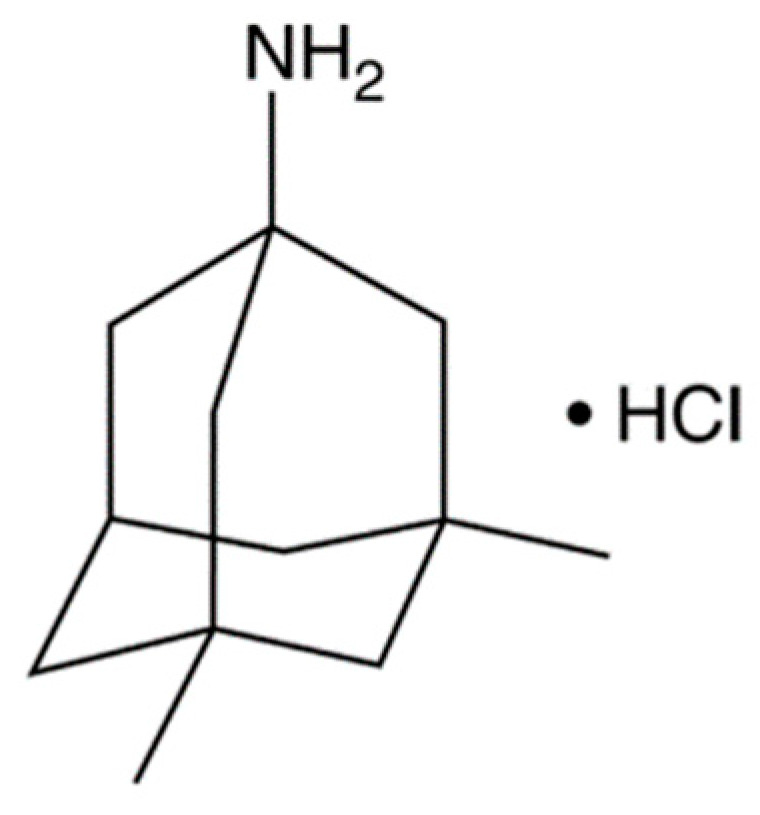
Chemical structure of memantine hydrochloride.

Memantine, a trade name for 1-amino-3,5-dimethyladamantane hydrochloride (Figure 1), is a low-affinity N-methyl-D-aspartate (NMDA) receptor antagonist that is used as the gold standard in the symptomatic treatment of moderate to severe stages of Alzheimer’s disease (AD) [1]. Nevertheless, attaining optimal therapeutic effects necessitates the maintenance of memantine concentrations within the therapeutic window, which ranges between 70 and 150 ng/mg in the plasma [2].

Memantine was approved by the European Medicines Agency (EMA) in 2002 and by the Food and Drug Administration (FDA) in 2003. It is currently the only approved NMDA receptor antagonist. This active pharmaceutical ingredient (API) functions by acting upon several histopathological changes identified in AD patients [3], namely by limiting the formation of neurofibrillary tangles, reducing microglial activation [4], lowering amyloid-β peptide levels [5], and improving patients’ cognition, global functioning behavior, and dementia stage [6,7].

The current memantine dosage forms are available as oral pills, oral solutions, and transdermal patches. With the progression of AD, the conventional ways of drug administration are a challenge: Patients often remove the patches and have swallow-related problems that compromise oral administration. As a consequence, the correct memantine dosage can hardly be guaranteed. Many drugs used to treat neurological disorders face challenges that limit their therapeutic effectiveness, such as restricted access to the brain due to biological pharmacokinetics. Nanoparticles (NPs) are a potential strategy to minimize adverse drug reactions and enhance medication safety. NPs can improve the therapeutic efficacy of drugs by protecting them from degradation while enhancing their bioavailability [8]. Additionally, their surface could be functionalized for a targeted administration, overcoming the biological barrier and reducing the undesired side effects of the unspecific distribution over the different tissues [9].

Quantification of the drug concentration is required to optimize therapeutic interventions in AD patients undergoing memantine treatment. For instance, in cases where patients exhibit subtherapeutic memantine concentrations, dosage adjustments can be made to ensure adequate drug exposure and enhance cognitive benefits. Conversely, patients with elevated memantine levels may be at increased risk of adverse reactions, warranting dose reductions to mitigate potential toxicity [10,11]. That way, the analytical method must be adequately validated to ensure a reliable quantification of the memantine encapsulated within the NPs.

Memantine is not yet available to be encapsulated in NPs for clinical use. For memantine, NP-based drug delivery systems are still preliminary in the experimental and research stages.

A variety of analytical methods have currently been developed for the quantification of memantine, including liquid chromatography–mass spectrometry (LC–MS) [12,13] and high-performance liquid chromatography (HPLC) with ultraviolet (UV) [14,15,16] or fluorescence detection [17,18]. Table 1 summarizes the advantages and disadvantages of these methods. However, none of these analytical methods for memantine quantification have been applied to determine memantine encapsulation within NPs.

Memantine cannot be directly detected using UV light because it has no native chromophores as part of its chemical structure [19]. Therefore, chemical derivatization is necessary to improve memantine detectability and separation performance by HPLC. The memantine-free base is extremely basic and lipophilic, meaning it binds to derivatization agents via ionic interaction with its primary amine group [20]. A few authors have reported pre-column derivatization of this API using such chemical derivatization agents as dansyl chloride [18,21,22], o-phthalaldehyde [23,24], 4-(4,5-diphenyl-1H-imidazol-2-yl)benzoyl chloride [25], and 9-fluorenylmethyl chloroformate (FMOC) [14,15,20,26,27]. FMOC is commonly used as a derivatizing agent since it reacts rapidly with most primary and secondary amines in alkaline buffers. Based on those previously published methodologies that use FMOC as a derivatization agent, only one work presents the optimization of the pre-column derivatization process, and this work is a HPLC method with fluorescence detection. This provided the impetus to develop and optimize the UV–HPLC method with pre-column derivatization of memantine hydrochloride using FMOC, searching for simple, rapid, precise, and accurate quantification of memantine.

Therefore, this work aimed to develop and validate the first HPLC method to determine the encapsulation efficiency (EE) of memantine incorporated in lipid NPs and its release over time in phosphate-buffered saline (PBS) to mimic physiological conditions, as it has a physiological pH of 7.4 at 37 °C and contains different electrolytes found in serum. In addition, our method entails complete optimization of the pre-column derivatization reaction in terms of the molar ratio of FMOC reagent to memantine, pH of borate buffer, and temperature and time of reaction.

The method was further validated in terms of suitability, specificity, linearity, sensitivity, precision, accuracy, and robustness per the International Conference on Harmonisation of Technical Requirements for Registration of Pharmaceuticals for Human Use (ICH) guidelines [28].

## 2. Results and Discussion

### 2.1. Optimization of Derivatization Method

**Figure 2:**
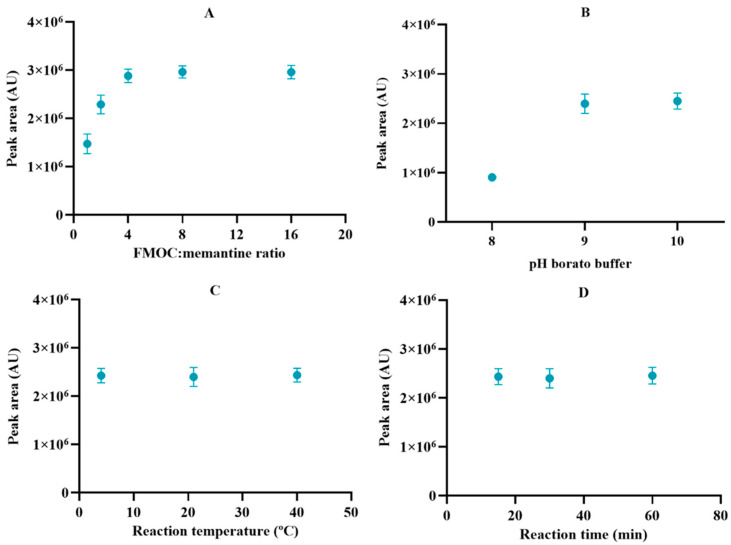
Optimization of the derivatization reaction condition in terms of (**A**) molar ratio of FMOC: memantine, (**B**) pH of borate buffer, (**C**) reaction temperature, and (**D**) reaction time. Results represent the mean ± SD, n = 3.

Since memantine barely absorbs in the UV spectrum, the method to detect this molecule relies on its derivatization with a chromophore. The derivatization method was optimized using the molar ratio of FMOC reagent to memantine, pH of borate buffer, and temperature and time of derivatization reaction as manipulated parameters (Figure 2). The results demonstrated that memantine was quantitatively derivatized under a molar ratio of FMOC to memantine above 4:1 and borate buffer at a pH higher than 9. No significant differences (*p* > 0.05) were observed between the distinct conditions of reaction time and temperature. Hence, the optimal conditions were found to be a molar ratio of FMOC to memantine of 8:1 (to ensure that there are enough molecules of FMOC in solution), borate buffer pH 9, and 30 min of reaction time at room temperature (approx. 21 °C), followed by incubation at 4 °C until the sample injection. These conditions were then used to quantify memantine in the remaining experiments.

### 2.2. Method Validation

The chromatographic conditions were reproduced when HPLC detection and suitability were checked. Derivatized memantine was detected at 11.393 ± 0.003 min after injection. The %RSD of the peak area was 3.9%, while the %RSD of retention time was 0.03%, thus indicating system suitability, as shown in Table 2.

**Figure 3:**
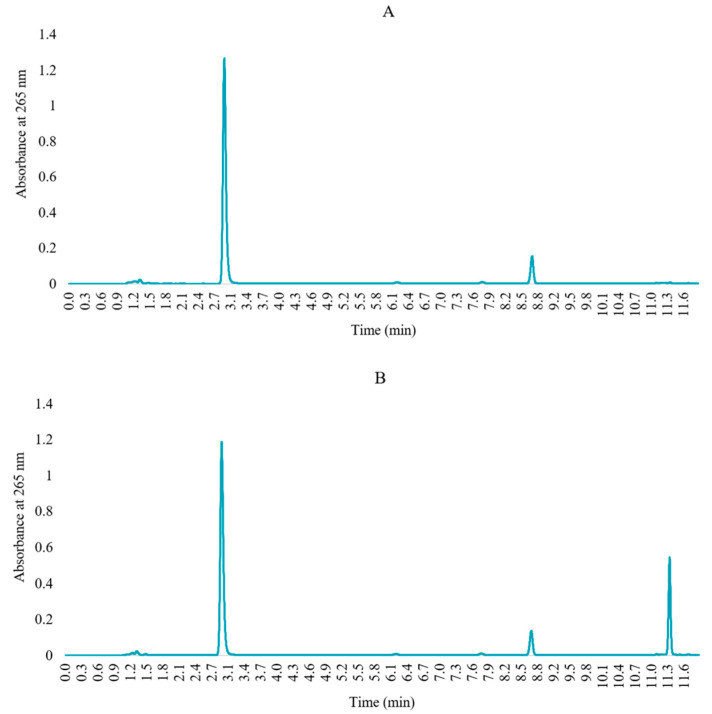
Chromatograms of (**A**) blank solution (FMOC in acetonitrile) and (**B**) derivatized memantine solution.

Specificity was determined by comparing the memantine target concentration solution and blank solution chromatograms. FMOC exhibits reactivity towards water, and FMOC-OH elutes in the middle of the chromatogram after hydrolysis and decarboxylation. At high levels, it can overlap with the memantine chromatogram, thus compromising its quantification [29]. Figure 3 indicated that chromatograms of a blank solution do not coincide with the chromatogram of derivatized memantine and show no exogenous interference upon the retention time, which unfolds the specificity of the developed HPLC method.

**Figure 4:**
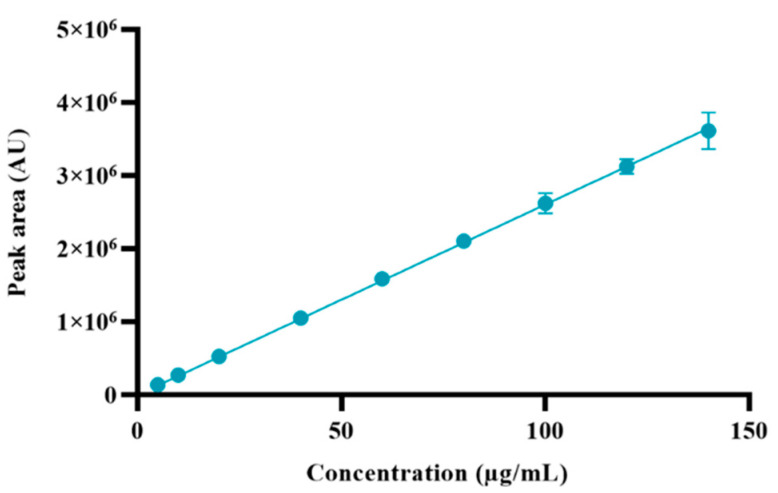
Calibration curve of derivatized memantine. Results represent the mean ± SD, n = 3.

The method linearity was investigated (Table 3) and described in terms of a calibration curve (Figure 4). The calibration curve was constructed using the peak area of derivatized memantine versus the concentration of memantine, and its linearity was determined using the linear regression analysis. The results demonstrate an excellent linear relationship between concentration and peak area within the tested range (5–140 µg/mL), which is confirmed by the slope of the linear calibration curve and the high coefficient of determination: R^2^ = 0.9999.

Sensitivity was provided by LOD and LOQ, which estimated values of 3.45 μg/mL and 10.45 μg/mL, respectively.

Intraday precision and the accuracy of memantine at three different concentration levels are depicted in Table 4. The %RSD of the concentration recovered for intraday and interday precision at all concentration levels was 3.6–4.6% and 4.2–9.3%, respectively, thus, within the acceptance criteria values. The method developed entails good precision, reliability, and reproducibility. The accuracy expresses closeness to the theoretical value regarding complete recovery. The mean recoveries were within the acceptance criteria values, except for interday accuracy at 120 μg/mL), thus representing high recovery values.

The robustness of the analytical method was evaluated by assessing the effect of slight variations in HPLC conditions, such as changes in detection wavelength, flow rate, column temperature, and injection volume. These results are summarized in Table 5. It was observed that a change in flow rate (±0.1 mL/min) or in detection wavelength (+3 nm) significantly affected recovery (*p* < 0.05), while the remaining changes in method conditions did not significantly affect the memantine peak area. Regarding retention time, no modifications to the method led to significant changes in retention time (*p* > 0.05). Therefore, one concluded that the proposed method is reliable and robust under those conditions.

**Figure 5:**
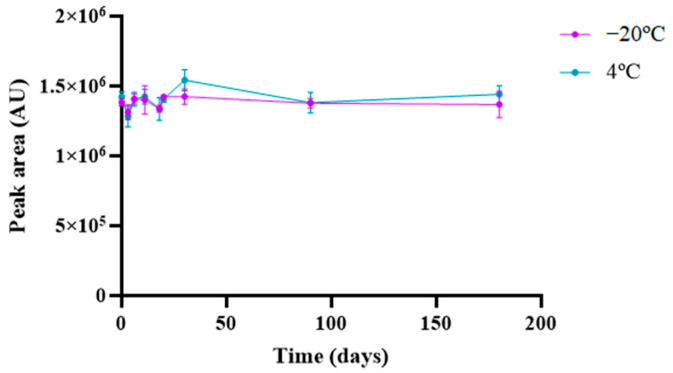
Stability analysis of memantine at 4 °C and −20 °C for 6 months. Results represent the mean ± SD, n = 3.

The long-term stability of memantine was evaluated at 1 mg/mL under different storage temperatures. After storage at 4 °C and −20 °C for six months, no significant degradation was observed over time (*p* > 0.05), as emphasized in Figure 5; this unfolds memantine stability at the noted conditions.

### 2.3. Applicability of the Method on a Nanoformulation: Evaluation of Encapsulation Efficiency

The lipid NPs were obtained with a mean particle size of less than 200 nm, which is essential for systemic administration and blood–brain barrier penetration [30]. PdI was about 0.2 for all formulations, showing no evidence of aggregation and an appropriate size distribution. Although this is not the ideal value for PdI, this type of distribution is typical in lipid NPs produced by high shear homogenization and ultrasonication, as the production parameters hinder the production of NPs with a homogeneous size distribution. The lipid NPs have ZP values high enough to ensure good stabilization of the NPs and prevent NPs from aggregating. The presence of memantine did not change the ZP of either SLNs or NLCs (*p* > 0.05).

The determination of the EE of memantine in NPs was performed indirectly, where the free memantine was completely separated from the lipid NPs. The EE of memantine within both types of NPs was carried out using the validated HPLC method, and the results are presented in Table 6. The EE was found to be 49 ± 1% and 46 ± 6% for SLNs and NLCs, respectively. This method proved to be suitable for the precise quantification of memantine in a short period of time.

## 3. Materials and Methods

### 3.1. Materials

Memantine hydrochloride (MW 215.77 g/mol, purity ≥ 98%) was obtained from Sigma-Aldrich (Aldrich, Steinheim, Germany). FMOC (MW 258.70 g/mol) was obtained from TCI Chemicals Europe (Zwijndrecht, Belgium). Acetonitrile (≥ 99%, HPLC) and trifluoroacetic acid (TFA, ≥ 99.9%) were obtained from VWR Chemicals (Radnor, PA, USA). Phosphate-buffered saline (PBS), pH 7.4 (10 mM phosphate buffer, 2.7 mM potassium chloride, and 137 mM sodium chloride, Sigma-Aldrich) was prepared in ultrapure water, purified, and obtained from Mili-Q equipment (Milli-Q Academic, Millipore, Guyancourt, France). Borate buffer was prepared in ultrapure water with *o*-boric acid (MW 61.83 g/mol) and di-sodium tetraborate decahydrate (MW 381.37 g/mol), both obtained from VWR Chemicals (Radnor, PA, USA). The buffers and samples were filtered using a membrane filter (0.22 μm pore size, from Restek, Bellefonte, PA, USA) before HPLC injection.

For NP syntheses, Pluronic^®^ F-127 (MW 12,600 g/mol) was obtained from Sigma-Aldrich (Aldrich, Steinheim, Germany), glyceryl palmitostearate (Precirol^®^ ATO 5) was kindly provided from Gattefossé (Saint-Priest, France), and medium-chain triglycerides (Miglyol^®^ 812) were a kind gift from Acofarma (Madrid, Spain).

### 3.2. Chromatographic System and Conditions

HPLC analysis was performed with a Waters (Milford, MA, USA) Alliance HPLC system (model 2695) equipped with a photodiode array detector (model 2998). The chromatographic separations were performed using a C18 Cortecs column (4.6 × 150 mm, 2.7 μm) (Waters, Milford, MA, USA) coupled to a C18 Cortecs VanGuard (3.9 × 5 mm, 2.7 μm). Ultrapure water with 0.02% TFA (A) and acetonitrile (B) were used as mobile phases. Derivatized memantine was separated using the following gradient: 0–5 min (50% B); 5–10 min (50–90% B); 10–12 min (90% B); 12–15 min (90–50% B); 15–20 min (50% B) (% in volume). The mobile phase flow was set at 1 mL/min, and the injection volume at 20 µL. The column temperature was set at 35 °C, and samples were preserved at 4 °C. The chromatograms were recorded at a wavelength of 265 nm. Memantine was identified by comparing the retention times of the derivatized memantine solution with the blank solution (the derivatization solution without memantine). Samples were injected in duplicate.

### 3.3. Derivatization Procedure

**Figure 6:**
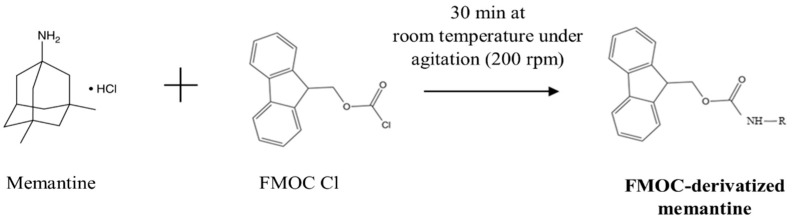
Reaction scheme of the derivatization of memantine with FMOC.

To detect memantine using UV, pre-column derivatization of memantine with FMOC-Cl (as illustrated in Figure 6) was applied based on previously developed methods [15], with modifications regarding the volume and molar concentration of the reagents that compose the derivatization reaction, as well as the time and temperature of the reaction. For memantine derivatization, 250 μL of memantine solution was used in PBS, an equal volume of 50 mM borate buffer (pH 9), and a predetermined volume of 10 mM FMOC solution in acetonitrile (corresponding to a molar ratio 8:1 of FMOC reagent to memantine). The mixture volume was completed with acetonitrile up to a total reaction volume of 1.0 mL. This mixture was incubated under stirring (200 rpm) in the shadow, followed by incubation at 4 °C prior to HPLC injection.

### 3.4. Optimization of Derivatization Procedure

The derivatization reaction was optimized to improve the specificity and accuracy of the method, using the molar ratio of FMOC reagent to memantine (ranging from 1 to 16), borate buffer pH (pH 8, 9, and 10), reaction temperature (4 °C, room temperature (approx. 21 °C), and 40 °C), and reaction time (15, 30, and 60 min) as manipulated conditions. Experiments were performed in triplicate for each condition.

### 3.5. Method Validation

The UV–HPLC method developed was validated according to the ICH guidelines (ICH Q2(R1)—Validation of Analytical Procedures: text and methodology) concerning suitability, specificity, linearity, sensitivity, precision, accuracy, and robustness [28]. Long-term stability studies of memantine under different storage temperatures were also conducted.

The acceptance criteria to validate the method were defined as RSD ≤ 10 and a percentage of recovery between 90 and 110. For linearity, the acceptance criterion was defined as R^2^ > 0.9995.

#### 3.5.1. Suitability

Suitability is the ability of an analytical system (HPLC in this case) to produce an adequate chromatographic peak concerning chromatographic quality assessment parameters (peak area, retention time, and system repeatability) and exhibit reproducibility. The system suitability was determined by injecting five replicates of the memantine solution (100 µg/mL).

#### 3.5.2. Specificity

This test aims to check the capability of the analytical method to quantify and unequivocally differentiate the API from the other expected components in the sample. Specificity was evaluated by independently comparing the chromatograms of the derivatized memantine solution (100 µg/mL) and blank solution (the derivatization solution without memantine). The blank solution was produced using the same protocol as the derivatized memantine solution but replacing the amount of memantine solution with PBS. The blank solution was also analyzed to ensure no peaks overlapped with the derivatized memantine peak.

#### 3.5.3. Linearity

The linearity was determined by designing calibration curves of derivatized memantine. The calibration curves were built using the square linear regression analysis of the peak area obtained as a function of the concentration of the derivatized memantine at several preset concentrations (ranging from 5 to 140 μg/mL, corresponding to 5–140% concentration of the API target concentration, respectively). The calibration curves were obtained in triplicate from three independent memantine solutions; their linearity was evaluated using the coefficient of determination (R^2^).

#### 3.5.4. Sensitivity

The sensitivity of the method was ascertained using the limit of detection (LOD) and limit of quantification (LOQ). The lowest amount of the analyte that can be detected, but not necessarily quantitated, under specified experimental conditions, is known as the LOD, whereas the LOQ is the lowest amount of analyte that can be quantitatively determined with precision and accuracy under specified experimental conditions. Both the LOD and LOQ were defined based on the standard deviation (SD) of the response and the slope of the memantine calibration curve, according to the following equations, respectively:

(1) $$\mathrm{LOD}=3.3\;\frac{\sigma }{S}$$

(2) $$\mathrm{LOQ}=10\;\frac{\sigma }{S}$$

where σ corresponds to the SD of the response, and S is the slope of the calibration curve.

#### 3.5.5. Precision and Accuracy (Intraday and Interday)

Precision tests aim to assess the degree of agreement between results obtained by applying the same method under the same conditions in different instances (intraday and interday). The accuracy represents the agreement between the concentration obtained by the analytical methodology and that actually present (theoretical concentration).

For the intraday variation study, five samples of three memantine concentrations, i.e., 80, 100, and 120 µg/mL (80, 100, and 120% of the memantine target concentration, respectively), were prepared and analyzed on the same day. For the interday variation study, a set of the same memantine concentrations was prepared every day for five days, and each set of samples was determined on the same day.

The peak area values were obtained from the chromatograms, and sample concentration and recovery percentage were determined. The precision and accuracy of the method were assessed using relative standard deviation (RSD) (Equation (1)) and recovery percentage (Equation (2)), respectively, of the intraday and interday variations, according to:

(3) $$\mathrm{RSD}\;(\%)=\frac{\mathrm{SD}\;\mathrm{of}\;\mathrm{recovery}}{\mathrm{Mean}\;\mathrm{of}\;\mathrm{recovery}\;}\times 100$$

(4) $$\mathrm{Recovery}\;(\%)=\frac{\mathrm{Measured}\;\mathrm{concentration}}{\mathrm{Theoretical}\;\mathrm{concentration}\;}\times 100$$

#### 3.5.6. Robustness

Robustness indicates the ability of an analytical method to generate essentially unchanged results irrespective of when small but deliberate changes in method experimental parameters. Based on our optimized method conditions, the effect caused by the following variations was evaluated toward the robustness of the proposed chromatographic method: detection wavelength (±3 nm), flow rate (±0.1 mL/min), column oven temperature (±5 °C), and injection volume (±10 µL).

Five samples of the memantine target concentration (100 µg/mL) were accordingly injected into the chromatographic system for each condition. The method robustness was assessed from the %RSD of the mean peak area and recovery of derivatized memantine.

#### 3.5.7. Stability Studies

Loss of API original features may occur because they are susceptible to decay during storage, which is promoted by minor changes in laboratory conditions such as temperature, humidity, or light. Long-term stability studies were thus carried out for 6 months to assess memantine stability (1 mg/mL) under different storage temperature conditions (4 °C and −20 °C). At predetermined time points, HPLC was used to analyze an aliquot of the samples. The derivatization procedure was performed immediately before HPLC injection. Experiments were performed in triplicate for each temperature and then compared to fresh solutions.

### 3.6. Preparation and Characterization of Memantine-Loaded NPs

Two different lipid NPs were tested in this work for memantine encapsulation: Solid lipid nanoparticles (SLNs) and nanostructured lipid carriers (NLCs).

SLNs were prepared using 250 mg of solid lipid (Precirol^®^ ATO 5), while NLCs were produced using 175 mg of solid lipid (Precirol^®^ ATO 5) and 75 mg of liquid lipid (Miglyol^®^ 812). The water phase comprised 2.175 mL of 10% water solution of Pluronic^®^ F-127 for both SLNs and NLCs. Lipid NPs were produced by combining the shear homogenization and the ultrasonication methods. First, the lipid phase was heated to at least 80 °C and allowed to melt completely. At the same time, the aqueous phase was heated at the same temperature. Then, the lipid phase was dispersed in the aqueous phase. An emulsion was obtained in a high-shear mixing device, Ultra-Turrax T25 (Janke and Kunkel IKA-Labortechnik, Staufen, Germany) for 1 min at 12,000 rpm to form the NPs, and then submitted to 15 min of ultrasonication at 80% amplitude and ultrasonic frequency of 24 kHz (UP400S ultrasonic processor, Hielscher, Berlin, Germany) to reduce the size of the NPs. Finally, NPs were left to cool down at room temperature (20–25 °C), allowing the lipid crystallization. For memantine encapsulation, 10 mg of memantine was incorporated in the lipid phase before melting and prepared according to the established conditions of the procedure.

The free amount of memantine was separated from memantine-loaded NPs using a gravity method (PD MiniTrap^TM^ G-10—2.1 mL Sephadex G-10, from GE Healthcare, Chicado, IL, USA) that separates molecules based on the differences in their size. The memantine-loaded NPs were diluted (1:10) and applied to the column (0.5 mL). The column was eluted with 1 mL of PBS for NP recovery and then eluted with 6 mL of PBS for free memantine recovery. The amount of free memantine was quantified by measuring the peak area of the supernatant by HPLC after FMOC derivatization using the method described herein. The free memantine concentration is calculated using the calibration curve obtained for derivatized memantine.

EE and loading capacity (LC) were determined indirectly by calculating the amount of free memantine (non-encapsulated) in the aqueous phase of NP dispersions as described in the following equations:

(5) $$\mathrm{EE}\;(\%)=\frac{\mathrm{Mass}\;\mathrm{of}\;\mathrm{memantine}\;\mathrm{in}\;\mathrm{formulation}-\mathrm{mass}\;\mathrm{of}\;\mathrm{free}\;\mathrm{memantine}}{\mathrm{Mass}\;\mathrm{of}\;\mathrm{memantine}\;\mathrm{in}\;\mathrm{the}\;\mathrm{formulation}}\;\times \;100$$

(6) $$\mathrm{LC}\;(\%)=\frac{\mathrm{Mass}\;\mathrm{of}\;\mathrm{memantine}\;\mathrm{in}\;\mathrm{the}\;\mathrm{formulation}-\mathrm{Mass}\;\mathrm{of}\;\mathrm{memantine}\;}{\mathrm{Mass}\;\mathrm{of}\;\mathrm{lipids}\;\mathrm{in}\;\mathrm{the}\;\mathrm{formulation}}\;\times \;100$$

NPs were characterized regarding their mean size, polydispersity index (PdI), and zeta potential (ZP) by Dynamic Light Scattering (DLS) using a Malvern ZetaSizer Nano ZS (Malvern Instrument, Malvern, UK) at 25 °C. Before each measurement, the samples were diluted in ultrapure water (1:100) to generate suitable scattering. Results were obtained by calculating the average of 3 measurements of 15 runs each.

### 3.7. Statistical Analysis

Data are expressed as the mean ± SD. Statistically significant differences were evaluated by a two-tailed Student’s *t*-test, considering any *p*-value below 0.05 (*p* < 0.05) as statistically significant; meanwhile, highly significant differences were any *p*-value below 0.01 (*p* < 0.01).

## 4. Conclusions

In this work, we developed the first simple, accurate, and precise UV–HPLC method to determine the EE of memantine hydrochloride in lipid NPs. Additionally, this study reached quantitative derivatization with FMOC via pre-column derivatization. Different conditions were tested in attempts to optimize the derivatization reaction. The best conditions for memantine derivatization included an 8:1 molar ratio of FMOC to memantine, a pH 9 of borate buffer at room temperature (ca. 21 °C), and a reaction time of 30 min, followed by incubation at 4 °C until injection. The pre-column derivatization with FMOC was successfully achieved through a simple reaction, and memantine detection was obtained without excipient interference.

Comparing our method with the previously used methods for detecting and quantifying memantine using UV–HPLC- detection, we present similar precisions. Regarding sensitivity, our linear range was higher than those previously published, as well as our LOD and LOQ values. Our chromatographic method was thoroughly validated per the ICH Q2(R1) guidelines in terms of suitability, specificity, linearity, sensitivity, precision, accuracy, and robustness—and proved acceptable for all such criteria.

A greenness study was performed to evaluate the impact of 12 principles of green analytical chemistry (GAC) in our method, using the Analytical GREEnness calculator [31]. Analytical GREEnness calculator software is freely accessed software available online (https://git.pg.edu.pl/p174235/AGREE, accessed on 29 July 2024).

**Figure 7:**
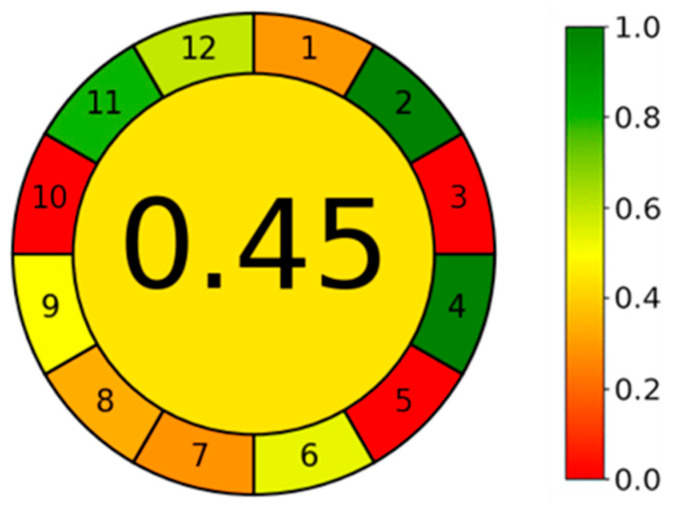
Pictogram of the HPLC method in Greenness study using the Analytical GREEnness calculator.

The results are presented in the pictogram in Figure 7. The HPLC procedure involves external sample treatment with a reduced number of steps (the derivatization of memantine) (principle 1) and the use of 20 μL of the sample (principle 2). The procedure is an offline measurement (principle 3) and involves one step, the derivatization (principle 4). The procedure is not automated or miniaturized (principle 5), although derivatization agents are involved in the analysis (principle 6). Analytical wastes include 1 mL of sample and 20 mL of the mobile phase (7.6 mL A and 12.4 mL B) (principle 7). One analyte is determined in a single run, and the throughput is five samples analyzed per hour, based on the retention time of 11 min (principle 8). Liquid chromatography is the analytical technique used (principle 9). Any reagents can be from bio-based sources (principle 10), and the procedure involves using a toxic reagent, the TFA (principle 11). TFA is considered toxic to aquatic life, and acetonitrile is considered inflammable (principle 12). The final score obtained was 0.45, calculated based on 12 principles of GAC. By default, we assumed that all 12 principles are equally important. Analyzing the pictogram, the GAC principles 3, 5, and 10 are quite low, and the principles 2 and 4 have an excellent performance.

Stability tests were conducted for up to six months to find the maximum storage temperature acceptable for samples. The method was demonstrated to be sensitive, precise, and accurate for the quantitative analysis of memantine in a concentration range suitable for application to nanoformulations designed as a potential strategy to minimize adverse memantine reactions and enhance their safety.

## Figures and Tables

**Table 1:** Comparison of the different analytical methods used to quantify memantine.

Method	Advantages	Disadvantages	Reference
LC–MS	High sensitivity (LOQ = 0.1 ng/mL) Matrix free from endogenous substance interference Low run time (retention time: 2.9 min)	Complexity of the sample preparation Need for an internal standard	[12]
	No peaks from endogenous compounds No interferences were apparent at the retention times of the analytes and internal standard High precision and accuracy (RSD < 5.3%) High sensitivity (LOQ = 1 ng/mL)	A twofold concentration step is needed The use of pure isotope-labeled internal standard High run time (retention time: 7.418 min)	[13]
HPLC—UV detection	High specificity (LOQ = 1.0 µg/mL) High precision (RSD < 0.08%) and accuracy (between 97.8 to 99.0%) Cost-effective	Pre-column derivatization required Speed of analysis (retention time: 6.54 min)	[14]
	High sensitivity (LOD = 0.36 µg/mL and LOQ = 1.09 µg/mL) High repeatability (RSDs < 6%)	Pre-column derivatization required	[15]
	No pre-column derivatization required Speed of analysis (retention time between 2.6 and 4 min)	High quantification calibration range (0.5–1.4 mg/mL) Low sensitivity (LOD = 0.035 mg/mL and LOQ 0.015 mg/mL) No method validation was performed	[16]
HPLC—fluorescence detection	More sensitive than UV detection Speed of analysis (retention time: 4.5 min) High sensitivity (LOD = 0.79 µg/mL and LOQ = 2.42 µg/mL High accuracy (between 94.8 and 119.4%)	Derivatization with a fluorescent probe is required Extensive and complex sample preparation	[17]
	More sensitive than UV detection High precision (RSD < 4.5%) High accuracy (between 94.3 and 100.7%) High sensitivity (LOD = 0.1 ng/mL) Simple sample preparation Excellent clean-up ability Requires negligible toxic organic reagent	Derivatization required	[18]

**Table 2:** Results of suitability parameters for the HPLC method.

Injection	Retention Time (min)	Peak Area (AU)	Recovery (%)
1	11.395	2,528,500	97.0
2	11.397	2,271,436	87.1
3	11.394	2,409,041	92.4
4	11.390	2,439,740	93.6
5	11.389	2,463,800	94.5
Mean	11.393	2,422,503	92.9
SD	0.003	95,185	3.7
RSD (%)	0.03	3.9	3.9

**Table 3:** Results of linearity parameters of the HPLC method.

Sample	Concentration (µg/mL)	Recovered Concentration (µg/mL)	Peak Area (AU)	Recovery (%)
1	5	5.38	140,164	107.5
2	10	10.47	273,057	104.7
3	20	20.23	527,249	101.1
4	40	40.33	1,051,297	100.8
5	60	60.91	1,587,880	101.5
6	80	80.83	2,107,151	101.0
7	100	100.58	2,622,031	100.6
8	120	119.91	3,125,719	99.9
9	140	138.63	3,613,751	99.0
Slope			26,068	
Coefficient of determination (R^2^)			0.9999	

**Table 4:** Precision parameter results for the HPLC method. Results represent the mean ± SD, n = 3.

Sample Con-Centration (µg/mL)	Measured Concentration (µg/mL)		Accuracy (Recovery, %)		Precision (%RSD)	
	Intraday	Interday	Intraday	Interday	Intraday	Interday
80	76.3 ± 3.5	72.0 ± 3.1	95.4 ± 4.4	90.0 ± 3.9	4.6	4.3
100	92.9 ± 3.7	93.6 ± 3.9	92.9 ± 3.7	93.6 ± 3.9	3.9	4.2
120	115.9 ± 4.2	106.1 ± 9.8	96.6 ± 3.5	88.4 ± 8.2	3.6	9.3

**Table 5:** Results of robustness parameters in the HPLC method. Results represent the mean ± SD, n = 3.

Parameter	Variation	Retention Time (min)	RSD (%) Retention Time	Mean Recovery (%)	RSD (%) Mean Recovery
Detection Wavelength (nm)	262	11.393 ± 0.003	0.03	92.8 ± 3.7	3.9
	265	11.393 ± 0.003	0.03	92.9 ± 3.7	3.9
	268	11.393 ± 0.003	0.03	83.2 ± 3.3	3.9
Flow Rate (mL/min)	0.9	11.798 ± 0.004	0.03	103.0 ± 4.1	4.0
	1	11.393 ± 0.003	0.03	92.9 ± 3.7	3.9
	1.1	11.030 ± 0.005	0.04	83.9 ± 3.5	4.1
Column Temperature (°C)	30	11.506 ± 0.004	0.04	92.7 ± 3.9	4.2
	35	11.393 ± 9.993	0.03	92.9 ± 3.7	3.9
	40	11.270 ± 0.004	0.03	90.7 ± 5.2	5.7
Injection Volume (µL)	10	11.393 ± 0.002	0.02	92.7 ± 3.8	4.1
	20	11.393 ± 0.003	0.03	92.9 ± 3.7	3.9
	30	11.391 ± 0.006	0.05	92.6 ± 3.7	4.0

**Table 6:** Characterization of lipid NPs before and after memantine encapsulation according to their size, PdI, ZP, EE, and LC (mean ± SD, n = 3). Data were analyzed using ANOVA. * and ** denote statistically significant differences (*p* < 0.05) and highly significant differences (*p* < 0.01), respectively.

Formulation	Mean Size (nm)	PdI	ZP (mV)	EE (%)	LC (%)
SLNs	101 ± 1	0.20 ± 0.01	−17 ± 4	-	-
SLNs–memantine	130 ± 6 *	0.26 ± 0.00 **	−12 ± 7	49 ± 1	2.0 ± 0.1
NLCs	133 ± 15	0.20 ± 0.02	−24 ± 3	-	-
NLCs–memantine	197 ± 16 **	0.19 ± 0.02	−10 ± 3 *	46 ± 6	1.9 ± 9.2

## Data Availability

All data are available upon request from the corresponding author.
